# Rhabdomyolysis in a Young Girl with Van Wyk-Grumbach Syndrome due to Severe Hashimoto Thyroiditis

**DOI:** 10.3390/ijerph15040704

**Published:** 2018-04-09

**Authors:** Alberto Leonardi, Laura Penta, Marta Cofini, Lucia Lanciotti, Nicola Principi, Susanna Esposito

**Affiliations:** 1 Pediatric Clinic, Department of Surgical and Biomedical Sciences, Università degli Studi di Perugia, Perugia 06132, Italy; alberto.leonardi88@gmail.com (A.L.); laura.penta@ospedale.perugia.it (L.P.); marta.cofini@gmail.com (M.C.); lucia.lanciotti@gmail.com (L.L.); 2 Università degli Studi di Milano, Milan 20122, Italy; nicola.principi@unimi.it

**Keywords:** Hashimoto thyroiditis, hypothyroidism, pseudoprecocious puberty, rhabdomyolysis, Van Wyk-Grumbach syndrome

## Abstract

*Background:* Autoimmune hypothyroidism (Hashimoto thyroiditis; HT) is the most common postnatal thyroid disease. Clinical manifestations of HT vary according to disease severity. Due to the pleiotropic effects of thyroid hormone, less common signs and symptoms of HT can occur, leading to a delay in diagnosis. *Case presentation:* A 9-year-old girl of Indian origin was admitted for a one-week history of widespread myalgia, fatigue, muscle weakness, difficulty walking, and a significant increase in weight (approximately 2 kg) without any changes in daily habits. The only relevant medical history was several intermittent vaginal bleeding episodes since four years of age. Breast development was consistent with Tanner stage 2 without pubic or axillary hair; while height and weight were at the 10th percentile and the 38th percentile; respectively. Bone age from a left wrist X-ray was delayed 1 year. Pelvic ultrasonography revealed a uterine body/neck ratio of >1 (pubertal stage) and multifollicular ovaries. Her external genitalia had a childlike appearance. Laboratory examinations showed an increased thyroid-stimulating hormone, decreased free thyroxine, and positive anti-thyroglobulin antibody titres, as well as elevation of creatine phosphokinase, myoglobin, lactate dehydrogenase, serum aspartate aminotransferase, hypercholesterolemia, and a basal serum prolactin near the upper limit of normal. Follicle stimulating hormone and estradiol were slightly and significantly elevated, respectively. Thyroid ultrasound showed an increased gland size with irregular echostructures and high vascularization. Levothyroxine replacement therapy led to complete normalization of clinical and laboratory findings, including rhabdomyolysis indices. No further vaginal bleeding episodes were reported. *Conclusion:* This case report highlights how various can be the clinical picture of HT in children, and how rare clinical manifestations can be the only signs of disease at presentation leading to delayed diagnosis and treatment. In this girl, a never-described association of Van Wyk-Grumbach syndrome and acute rhabdomyolysis in a young girl with previously unrecognized HT is described. The importance of recognizing the signs and symptoms of rare complications of HT in order to begin appropriate therapy is stressed.

## 1. Introduction

Autoimmune hypothyroidism (Hashimoto thyroiditis, HT) is the most common postnatal thyroid disease. In paediatrics, the global prevalence is estimated to be 1% to 2% with variations according to age, sex, ethnicity, and the presence of other autoimmune diseases or genetic syndromes [1,2]. HT is rare in children under 3 years of age and is more common in older children and adolescents. Females have a 4:1 predominance, and Caucasians suffer from HT more frequently than Blacks. Finally, HT is more commonly diagnosed in children with type 1 diabetes, coeliac disease, Addison disease, hypoparathyroidism, Down syndrome, Noonan syndrome, and Turner syndrome [1,2].

Clinical manifestations of HT vary according to disease severity. Initially, most cases present with only a slight enlargement of the thyroid that can go unnoticed or is not properly evaluated. Later, the most common signs and symptoms are bradycardia, delayed reflexes, myxoedema of the face and limbs, dry skin, fatigue, cold intolerance, and constipation. In the most severe cases, poor growth velocity and reduced school performance are common. However, due to the pleiotropic effect of the thyroid hormone, less common signs and symptoms of the disease can occur and, in some patients, it may be the major presenting clinical manifestations leading to a difficult or delayed diagnosis, as in the girl here described. [1,2]. In this patient, autoimmune hypothyroidism had an uncommon clinical course. It developed at an early age, presented initially with signs of isosexual incomplete precocious puberty, and was complicated by rhabdomyolysis. Diagnosis was delayed. Fortunately, substitutive therapy was effective, and rare manifestations of hypothyroidism were reverted.

## 2. Case Presentation

A 9-year-old girl of Indian origin presented to our paediatric clinic for a one-week history of widespread myalgia, fatigue, muscle weakness, and difficulty walking. Moreover, in the previous 15 days, she had a significant increase in weight (approximately 2 kg) without any changes in daily habits. Recent history was negative for trauma, infection, vigorous exercise, and drug use. The only relevant medical history was several intermittent vaginal bleeding episodes since 4 years of age, treated only with topical therapy and not associated with local trauma or accidental intake of hormonal drugs. With regard to family history, both the mother and brother had HT.

On physical examination, the girl was found to be in good general condition with normal psychological development. Vital signs were within normal range (body temperature 36.5 °C, respiratory rate 20 breaths/min, oxygen saturation 99% in air, blood pressure 100/60 mmHg), except for bradycardia (65 bpm; <2 standard deviation [SD] for age). Skin xerosis and an enlarged thyroid were found. Moreover, she had muscular weakness that almost prevented her from walking. Furthermore, passive mobilization and palpation of the muscles of the limbs caused pain. No signs of muscular pseudohypertrophy were found. Deep tendon reflexes were poor in all limbs. There were no cranial nerve deficits or cerebellar or meningeal signs.

Breast development was consistent with Tanner stage 2 without pubic or axillary hair, while auxological data were consistent with a height at the 10th percentile (−1.27 SD, mid parental height −2.47 SD) and weight at the 38th percentile according to the Tanner charts. Body mass index (BMI) was 19.65 kg/m^2^ (75th percentile). Bone age from a left wrist X-ray was delayed 1 year with respect to chronological age. Pelvic ultrasonography revealed a uterine body/neck ratio of >1 (pubertal stage) and multifollicular ovaries. Her external genitalia had a childlike appearance. 

Table 1 lists the results of laboratory examinations. Blood tests showed significant elevation of creatine phosphokinase (CPK), myoglobin lactate dehydrogenase (LDH), and aspartate aminotransferase (SGOT). Hypercholesterolemia (total cholesterol 294 mg/dL) and a basal serum prolactin near the upper limit of normal (20.08 ng/mL) were evidenced. Follicle stimulating hormone (FSH) and estradiol were slightly and significantly elevated, respectively. On the contrary, luteinizing hormone (LH) serum levels were very low. Other laboratory tests were unremarkable. She had no history of dark urine, and the urine examination performed in our clinic was negative. 

The presenting symptoms and laboratory data were compatible with acute rhabdomyolysis with no renal or cardiac involvement. In fact, in addition to the absence of specific symptomatology of these organs, both laboratory and instrumental tests were negative. Therefore, the clinical history was thoroughly investigated, and blood sampling was performed to exclude the most common causes of CPK elevation (i.e., viral or bacterial infections, trauma, exotoxin exposure, convulsive phenomena, or connective tissue disease).

**Figure 1:**
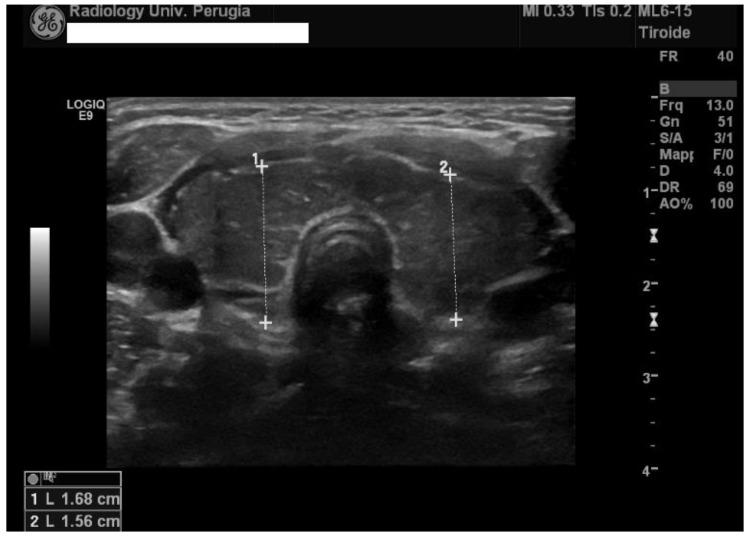
A thyroid ultrasound with evidence of an enlarged thyroid gland (total volume 10 mL) with non-homogeneous echotexture.

Taking into account the family history of autoimmune thyroiditis and the presence of suspicious symptoms (i.e., weight gain, goitre, xerotic skin, and bradycardia), the girl’s thyroid function was investigated with the following results: thyroid-stimulating hormone (TSH) 357 μIU/mL (normal range 0.34–5.60), free thyroxine (fT4) 0.26 ng/dL (normal range 0.54–1.24), and negative anti-thyroperoxidase (TPO) and positive anti-thyroglobulin antibody titres (Table 1). Thyroid ultrasound showed increased gland size with irregular echostructures and high vascularization (Figure 1). 

In relation to such findings, acute rhabdomyolysis with severe HT was diagnosed. Intravenous saline was given, and levothyroxine replacement therapy was started at a dose of 1.62 mcg/kg/day. One week after initiating therapy, there was complete normalization of rhabdomyolysis indices and resolution of all presenting symptoms. Even the elevation of cholesterol levels was attributed to thyroid disease, which normalized shortly after the initiation of substitution treatment (Table 1). No further vaginal bleeding episodes were reported after the diagnosis, and she had menarche at 11 years old. 

The resolution of vaginal bleeding after starting substitution therapy also confirmed the diagnosis of Van Wyk-Grumbach syndrome. In fact, according to this syndrome, the girl had precocious isosexual puberty with typical delayed bone age on wrist/hand X-ray and menarche with no signs of adrenarche. 

Six months after admission, with gradual dose adjustments of the levothyroxine, euthyroidism was achieved. One year later, she had significant auxological improvement: Her height reached the 19th percentile (−0.87 SD), and her height growth velocity was 7.99 cm/year (3.14 SD). Twenty months after levothyroxine therapy, her height was at the 32th (−0.48 SD) percentile with a height growth velocity of 10.02 cm/year (4.39 SD). Weight and BMI remained nearly unchanged. 

Management and publication of this case was approved by Ethics Committee of Azienda Ospedaliera Santa Maria della Misericordia, Perugia, Italy (2018_PED_CR_01). Both parents of the patient signed a written informed consent and the patient signed a written assent.

## 3. Discussion

This is a case of paediatric HT, as evidenced by clinical signs and symptoms suggesting hypothyroidism and laboratory findings of autoimmunity against the thyroid. However, this case has some peculiar characteristics. First of all, it is the first case of HT in which two rare complications of hypothyroidism, rhabdomyolysis and Van Wyk-Grumbach syndrome, were the most significant manifestations of the disease and were diagnosed simultaneously in the same patient. Moreover, this case is very instructive. It is a typical example of how the clinical presentation of acquired hypothyroidism may be different from what is expected and how diagnostic delay may lead to the development of severe clinical problems. 

In this girl, signs of isosexual incomplete precocious puberty, mainly vaginal bleeding, were the first relevant manifestations of HT. They were neglected for years and this led to the development of a more advanced and severe clinical picture, including a dominant sign of severe rhabdomyolysis, a very rare complication of HT with potentially dramatic consequences. Although hypothyroidism is generally associated with delayed puberty [1,2], incomplete precocious puberty can develop. Boys have precocious testicular enlargement without virilization and girls experience vaginal bleeding, breast development, and, sometimes, galactorrhoea [3]. Pelvic ultrasonography generally shows an enlarged uterus and ovarian cysts. Laboratory blood tests demonstrate increased serum TSH, low fT4, normal to elevated prolactin, oestradiol and follicle stimulating hormone (FSH), and a suppressed luteinizing hormone (LH) level. Characteristically, contrary to true precocious puberty, bone age is delayed [4,5,6,7,8]. Interaction of TSH with the FSH receptor leading to gonadal hyperstimulation is the most accepted hypothesis to explain incomplete precocious puberty [9]. Moreover, increased production of prolactin explains LH suppression, sensitization of the ovaries to circulating gonadotrophins, and acceleration of follicular maturation.

Most of the clinical, laboratory, and radiological findings in the present case were revealed when the patient was hospitalized. Moreover, the patient had other signs of HT, including increased serum anti-thyroglobulin antibody levels, increased thyroid volume, skin xerosis, and hypercholesterolemia. This led to the diagnosis of Van Wyk-Grumbach syndrome, a condition in which hypothyroidism is associated with variable functional and structural alterations of the ovarian structure secondary to excessive TSH production. This causes stimulation of FSH receptors, increased oestrogen production, polycystic degeneration, and, rarely, malignant tumour degeneration [10].

The most significant clinical manifestation of the disease when HT was diagnosed in this patient was clinically evident rhabdomyolysis. Neuromuscular and musculoskeletal manifestations are common in hypothyroid patients [11]. Muscle involvement typically manifests as polymyositis-like myopathy with muscle weakness and increased serum levels of muscle enzymes such as CPK, SGOT, and LDH [12,13]. Consequently, breakdown of striated muscles with leakage of the intracellular muscle constituents into the circulation and extracellular fluid is common in HT patients. Several factors are thought to induce muscle damage, including low ATP turnover in the skeletal muscle, low myosin ATPase activity, poor contractility of the actin-myosin unit, and structural changes in muscle fibres that switch from the fast-twitch type II to the slow-twitch type 1 [14]. In most of the cases, rhabdomyolysis is minimal and remains asymptomatic. However, in some patients in which CPK levels are greater than 5000 UI/L [15], muscle destruction can result in dramatic clinical consequences coagulation [16], including acute renal failure, severe electrolyte imbalance, and disseminated intravascular. 

Fortunately, clinically evident rhabdomyolysis due to hypothyroidism is rare, particularly in children. Less than 10 cases have been described [17,18,19,20]. In this patient, even though myalgia, muscle weakness, and difficulty walking were evident, rhabdomyolysis was of moderate severity because CPK levels, although very high, remained lower than 5000 UI/L, and no myoglobinuria was demonstrated. However, especially when rhabdomyolysis is the most significant presenting sign of hypothyroidism or is associated with other unusual signs and symptoms of this disease, diagnosis of the underlying endocrinological problem can be difficult and delayed. 

## 4. Conclusions

This is the first patient in whom two rare complications of hypothyroidism, Van Wyk and Grumbach syndrome and rhabdomyolysis, were the most significant manifestations of HT and were diagnosed simultaneously. As evidenced by the clinical history, both incomplete precocious puberty and rhabdomyolysis were rapidly responsive to substitutive therapy. Moreover, administration of levothyroxine eliminated all clinical manifestations of hypothyroidism, including reduction of height growth and weight gain velocity. This highlights the importance of recognizing signs and symptoms of rare complications of HT for beginning an appropriate therapy and avoiding severe clinical problems.

## Figures and Tables

**Table 1:** Laboratory results on admission and during follow-up from the time of initiation of levothyroxine therapy.

Test (Reference Interval)	0 Day	1 Week	1 Month	3 Months
TSH (0.340–5.600 µIU/mL)	357		6.37	1.49
fT4 (0.54–1.24 ng/dL)	0.26		1.00	0.89
Anti-TPO antibodies (0.0–9.0 UI/mL)	3.4			
Anti-thyroglobulin antibodies (0–4.9 UI/mL)	73.7			
FSH (1.6–9.6 mUI/mL)	10.2			
LH (1–11 mIU/mL)	0.10			
Estradiol (20–60 pg/mL)	86			
SGOT (<45 UI/L)	105	44		
LDH (225–450 UI/L)	748	496	436	
CPK (0–180 UI/L)	3395	392	170	
Myoglobin (14.3–65.8 ng/mL)	76	52		
Total cholesterol (95th percentile: 197 mg/dL)	294	209	156	

TSH: thyroid-stimulating hormone; fT4: free thyroxine; TPO: thyroid peroxidase; FSH: follicle stimulating hormone; LH, luteinizing hormone; SGOT: aspartate aminotransferase; LDH, lactate dehydrogenase; CPK, creatine phosphokinase.
